# In situ evaluation of stalk lodging resistance for different maize (*Zea mays* L.) cultivars using a mobile wind machine

**DOI:** 10.1186/s13007-019-0481-1

**Published:** 2019-08-20

**Authors:** Weiliang Wen, Shenghao Gu, Boxiang Xiao, Chuanyu Wang, Jinglu Wang, Liming Ma, Yongjian Wang, Xianju Lu, Zetao Yu, Ying Zhang, Jianjun Du, Xinyu Guo

**Affiliations:** 1 Beijing Research Center for Information Technology in Agriculture, Beijing, 100097 China; 2 Beijing Key Lab of Digital Plant, National Engineering Research Center for Information Technology in Agriculture, Beijing, 100097 China

**Keywords:** Failure wind speed, Cumulative lodging index, Maize (*Zea mays* L.), Mechanical properties, Lodging resistance, Wind machine

## Abstract

**Background:**

Stalk lodging is an impediment to improving profitability and production efficiency in maize. Lodging resistance, a comprehensive indicator to appraise genotypes, requires both characterization of mechanical properties in laboratory and investigation of lodging percentage in field. However, in situ characterization of maize lodging resistance still remains poor. The aim of this study was to develop an indicator, named cumulative lodging index (CLI), based on lodging percentages at different wind speeds for evaluating lodging resistance for different maize cultivars, and to evaluate the accuracy and reliability of this method.

**Results:**

Different cultivars showed different patterns of lodging percentage along with wind speeds. The failure wind speed (FWS) for maize ranged between 16 and 30 m s^−1^ across cultivars. The CLI differed between maize cultivars and showed favorable reliability (i.e. nRMSE of 5.38%). Mechanical properties of the third internode did not vary significantly between cultivars. Significant differences in the reduction index (RI) of wind speed sheltered by maize canopy were found between cultivars.

**Conclusion:**

Our findings implied that mobile wind machine is powerful in reproducing wind disaster that induce crop lodging. The newly-built CLI was demonstrated to be a more robust indicator than mechanical properties, FWS, and RI when evaluating lodging resistance in terms of both reliability and resolution. This study offers a new perspective for evaluating in situ lodging resistance of crops, and provides technical support for accurate identification of lodging-resistant phenotypic traits.

**Electronic supplementary material:**

The online version of this article (10.1186/s13007-019-0481-1) contains supplementary material, which is available to authorized users.

## Background

Maize (*Zea mays* L.) is one of the most important grain crops in the world, and its high productivity is essential for meeting the increasing demand for food, livestock, and bio-fuel. Maize stalk lodging is defined as the breakage at or below the ear [1], which makes up more than 60% of lodging cases in a dense population [2], resulting in a major issue in modern maize production. It causes annual yield losses of up to 75% [3–6], reduces grain quality [7], and increases costs to harvest [8]. In addition, the frequency of extreme wind speed is projected to increase with global warming in the extratropics [9] where major maize production area in China are included, increasing the potential risk of maize lodging. To reduce the vulnerability of maize production induced by extreme weather, understanding maize stalk lodging is essential to increase yield through cultivation optimization and genetic improvement.

Lodging resistance has long been a pivotal component in genetic gains in grain yield of cultivars for maize in the half past century across the world [10]. Extensive studies have been performed to quantify stalk lodging resistance in order to appraise genotypes in maize lodging accurately [11–13]. Lodging rate, the ratio of the number of lodging plants to the total number per unit area, has been demonstrated to be negatively related to stalk lodging resistance and regarded as the most direct way to evaluate stalk lodging resistance [14]. However, the field survey of stalk lodging for maize showed a great variation with an average coefficient of variation of 82% from a long-term experiment [11]. Instead of visual counting manually, unmanned aircraft system (UAS) has been proposed to measure the number of lodging plants and the lodging rate on a per-row basis, facilitating the high-throughput phenotyping for maize lodging [15]. However, visual counting of maize stalk lodging, which is normally used in field investigation to assess stalk lodging resistance in previous studies, are unreliable for quantitatively evaluating the lodging resistance of certain cultivars under different wind speeds because the stochastic expression of stalk lodging is significantly affected by diseases, insects, and wind [11, 16].

Stalk lodging resistance can also be qualitatively described with the measurement of morphologic traits, as several traits have been suggested to closely associate with stalk lodging resistance. For example, the resistance of maize to stalk lodging is positively related to basal internode diameter [17] and the ratio of internode diameter to length [3]. It is also negatively correlated with basal internode length [3] and the ear coefficient (i.e. ear height per plant height) [18]. The section modulus of the stalk has been demonstrated to be a highly predictive indicator of stalk strength with a correlation coefficient (R^2^) ranging from 0.73 to 0.80 [19]. At the anatomical level, the total area of vascular bundles in the peripheral layer, auxiliary axis diameter, and total area of vascular bundles are three parameters most related to stalk strength [20]. However, these studies were conducted by either correlating field-based visual count of lodging rate with agronomic traits, or linking stalk strength to morphological features under laboratory conditions.

Measuring stalk strength has proven reliable in assessing stalk lodging resistance in the absence of extreme weather. Stalk crushing strength was initially developed by measuring the force required to break a stalk using a hydraulic press [21]. Sibale et al. [22] measured rind puncture resistance using a modified electronic rind penetrometer to help select superior stalk strength for maize. Stalk flexural stiffness, a mechanical measurement inspired by a structural engineering beam theory, predicted 81% of the variation in stalk strength, whereas rind puncture resistance only explained 18% of the variation [23]. Although methods for measuring maize stalk strength have become increasingly convenient and efficient, they have all been performed under controlled laboratory conditions; therefore, the reliability may not be hold under field conditions.

An additional measurement for stalk lodging resistance is the mechanical model, which has been adopted to predict the specific stress under which stalk lodging occurs by using the failure wind speed (FWS). FWS, the wind speed at which the bending strength at a certain point along the stem was exceeded [24], provides an indicator for quantitatively describing the stalk lodging resistance. Accordingly, FWS was predicted to be 22.8 m s^−1^ for forest trees and 11.6 m s^−1^ for the canopy of cereals [25]. However, the accuracy of predictions largely depends on measuring many material properties and estimating probability distributions of bending moment, both of which require extensive investigations.

To determine FWS for evaluating stalk lodging resistance, the use of a wind tunnel is the most intuitive and practical method [26]. A portable wind tunnel was previously constructed to confirm its competence to quantitatively investigate the lodging process under various windy conditions and to evaluate the accuracy of existing theoretical models for plant withdraw [27]. This wind tunnel, however, cannot be used to simulate the effects of wind on maize lodging due to its limited wind speeds with a maximum of 8.5 m s^−1^ [27]. DuPont Pioneer has devised a mobile wind machine that can generate winds up to 45 m s^−1^ to assess stalk lodging under controlled wind conditions, facilitating the evaluation of a genotype for maize lodging [28]. However, few studies have been reported on quantifying FWS and evaluating lodging resistance for maize using an appropriate wind tunnel. The lack of data from well-controlled field experiments in terms of FWS and its related key phenotypic traits has restricted the reliability of evaluating lodging resistance for maize cultivars. Furthermore, there is little information on the comprehensive study of maize stalk lodging by combining wind simulation in situ and destructive measurement in lab.

In this study, we aim to build a controllable field wind machine in order to: (1) quantify the FWS for different maize cultivars; (2) introduce a new method to determine the lodging resistance at different wind speeds; (3) evaluate the lodging resistance under progressively increasing wind speed for different cultivars; (4) characterize the attenuation of wind speed for different cultivars; and (5) determine the relationship between lodging resistance and phenotypic traits.

## Results

### Controllable wind machine for use in the field

**Fig. 1:**
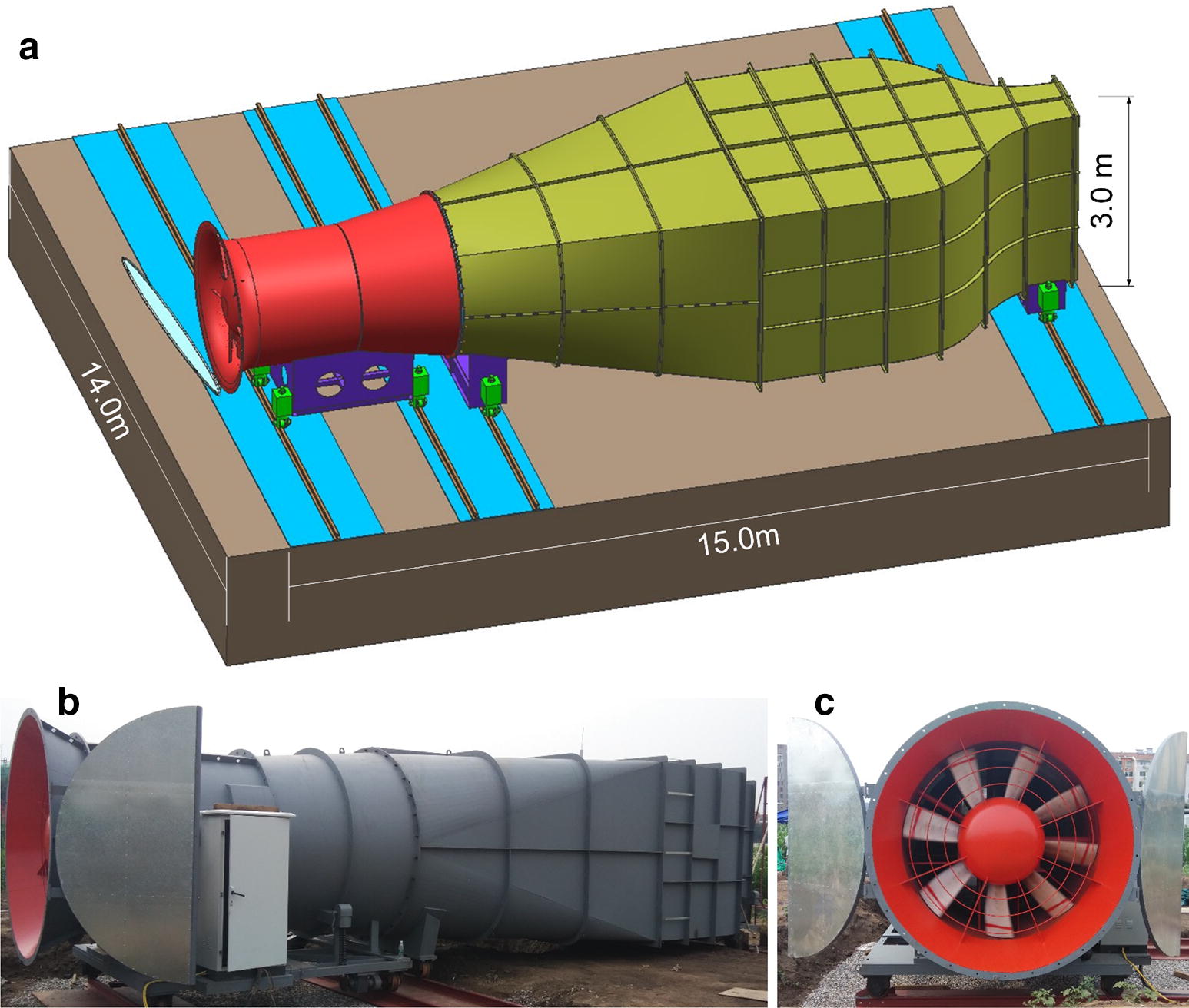
A three-dimensional diagram of wind machine (**a**), the main structure from side view (**b**), and the fan (**c**). The brown part in the diagram represents solid ground, and blue represents the rails. The red part denotes the high-speed fan, and the yellow denotes main structure including a settling chamber and a contraction area

To evaluate field crop lodging resistance, a mobile wind machine (Beijing Aerospace Yisen Wind Tunnel Engineering Technology Co., Ltd.) was built in the experimental field of the *Beijing Academy of Agriculture and Forestry Sciences* (39°56′N, 116°16′E, and 70 m altitude) in 2015 (Fig. 1). It is composed of a high-speed fan with a diameter of 2.8 m, a settling chamber (15 m long by 3 m tall), and a contraction area (6 m long by 4 m wide). The fan is able to generate a rate of flow up to 5500 m^3^ min^−1^ (equivalent to 35 m s^−1^), a maximum wind pressure from 800 to 900 Pa, a rated power of 130 kw, and a rotation rate of 990 rpm. The width and height of the wind outlet is 1 m and 3 m, respectively. The role of the settling chamber is to eliminate swirl and unsteadiness from the flow, and the purpose of the contraction area to make the flow more uniform. The wind machine can be moved along four parallel rails, which are 14 m long and are perpendicular to the flow direction, by manually rotating two handles. The fan can force air at wind speeds ranging from 0 to 35 m s^−1^. To ensure the safety of wind machine, a maximum of 30 m s^−1^ was set, which is slightly lower than the upper limit of 35 m s^−1^. The generated wind speed has an error of less than 5%, which is in the effective range of space and time. A testing process for XD20 in 2016 is given in the Additional file 1.

### Wind speed attenuation under natural conditions

**Fig. 2:**
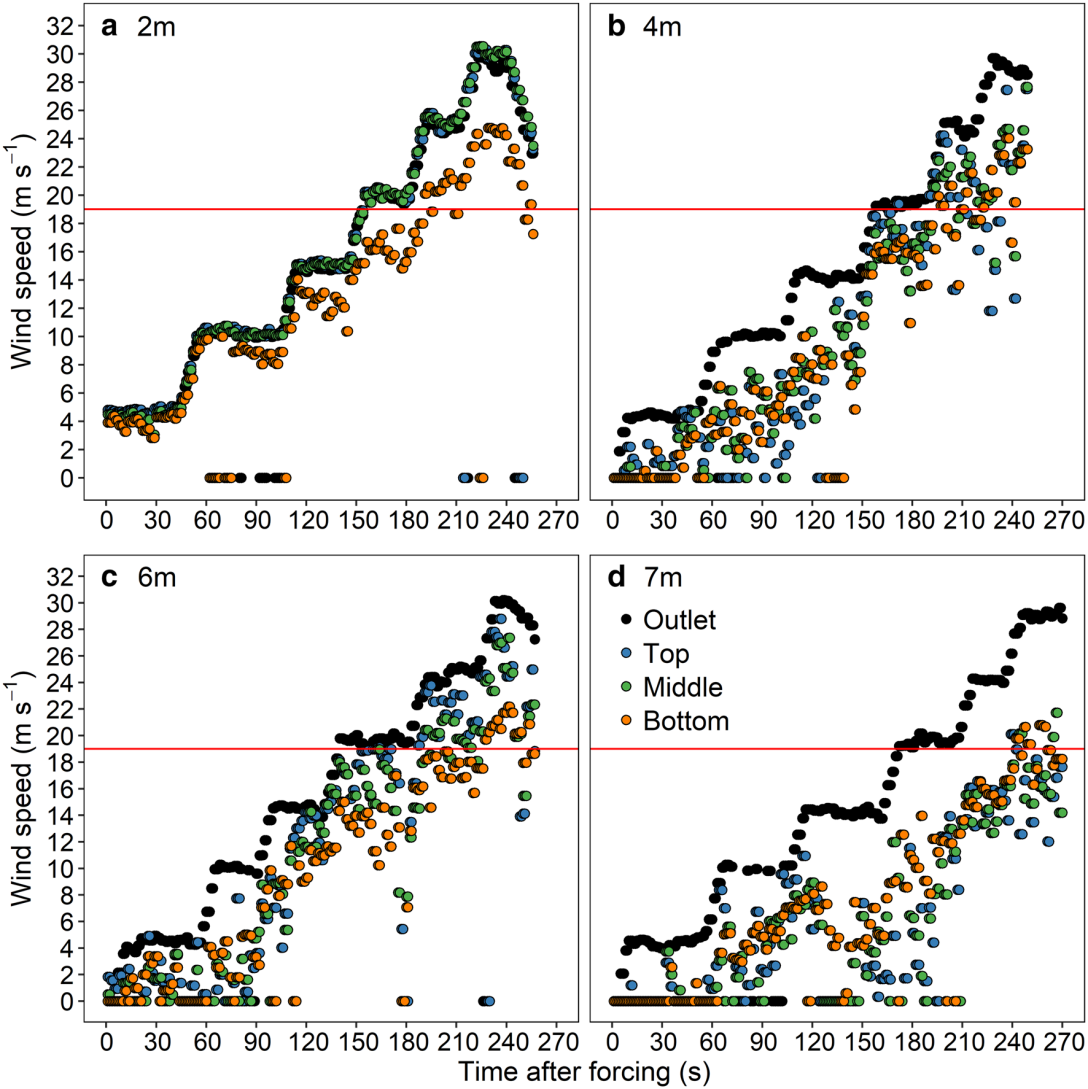
Measured wind speed at the outlet of wind machine, at the top, middle, and bottom layers of the holder when it was fixed at 2 m (**a**), 4 m (**b**), 6 m (**c**), and 7 m (**d**) away from the outlet. The red line represents an estimated FWS of 19 m s^−1^ from literature

Wind speed attenuated with the distance away from the outlet of the wind machine (Fig. 2). Due to the resistance created by the ground, wind speed above the ground had a greater reduction than other two layers for all distances. The overall reduction index in the bottom, middle, and top layers increased from 0.22, 0.02, and 0.02 at 2 m to 0.59, 0.65, and 0.66 at 7 m from the outlet, suggesting an increasing reduction of wind speed with increasing the distance away from the outlet. The wind speed at 7 m fell below 19 m s^−1^ when a maximum generated wind speed of 30 m s^−1^ was achieved. Therefore, a maximum row length of 7 m was determined for the wind machine tests.

### Mechanical properties and phenotyping traits

Table 1 shows the measured stalk mechanical properties and phenotyping traits of the six cultivars. Young’s modulus, maximum bending load, and maximum transverse displacement did not differ significantly across cultivars. Plant height was significantly higher for JK665, XY335, and XD20 than for ZD958. JK665, ZD958, and JK968 had the greatest number of leaves, while XY335 had the fewest. The leaf inclination angle of XD20 was found to be significantly higher than other cultivars. ZD958 had a significantly greater ear number per plant than other cultivars. The height of ear for XD20 was significantly greater than that of the other cultivars. Plant azimuthal deviation and ear length did not show significant differences across cultivars.

**Table 1:** Mechanical properties of internode at rank 3 of maize stem and phenotypic traits for six cultivars in 2016

Cultivars	Young’s modulus (10^3^ MPa)	Maximum bending load (N)	Maximum transverse displacement (mm)	Plant height (mm)	Plant azimuthal deviation (°)	Leaf number	Leaf inclination angle (°)	Ear number	Ear height (mm)	Ear length (mm)
ZD958	64.1 ± 8.1a	365.7 ± 25.7a	9.0 ± 0.8a	221.4 ± 12.4b	28.3 ± 4.0a	12.8 ± 0.6a	62.2 ± 2.8b	2.5 ± 0.3a	85.2 ± 6.4b	28.4 ± 1.9a
JD38	90.0 ± 1.4a	406.6 ± 6.7a	9.3 ± 0.4a	239.6 ± 4.5ab	29.0 ± 3.1a	11.4 ± 0.4ab	63.5 ± 1.7ab	1.4 ± 0.2b	88.7 ± 4.7b	27.6 ± 1.6a
XD20	67.0 ± 2.3a	366.6 ± 23.4a	8.1 ± 0.9a	256.0 ± 3.5a	22.5 ± 4.0a	11.6 ± 0.4ab	69.8 ± 0.6a	1.6 ± 0.2ab	120.1 ± 2.6a	26 ± 0.6a
XY335	98.2 ± 8.9a	421.6 ± 47.2a	8.1 ± 1.5a	259.0 ± 3.0a	20.2 ± 3.1a	10.2 ± 0.2b	63.9 ± 1.2ab	1.4 ± 0.2b	91 ± 3.2b	27.8 ± 1.8a
JK665	83.6 ± 13.7a	422.7 ± 70.7a	8.3 ± 0.7a	260.7 ± 3.5a	20.6 ± 2.9a	13.0 ± 0.3a	61.7 ± 1.6b	2.0 ± 0ab	90.2 ± 6.6b	32.2 ± 1.2a
JK968	85.3 ± 7.5a	432.0 ± 34.5a	8.1 ± 0.4a	236.8 ± 8.6ab	19.0 ± 1.3a	12.0 ± 0.3a	65.6 ± 1.7ab	1.6 ± 0.2ab	103.2 ± 9.5ab	30.6 ± 2a
P	ns	ns	ns	**	ns	**	*	*	**	ns

ANOVA was carried out for all cultivars. Absence of shared letters denotes a statistically significant difference (*P *= 0.05) between cultivars using Tukey test. Significance levels (** *P* < 0.01, * *P* < 0.05, and ^ns^ *P* ≥ 0.05) are given

### Failure wind speeds and cumulative lodging index

**Fig. 3:**
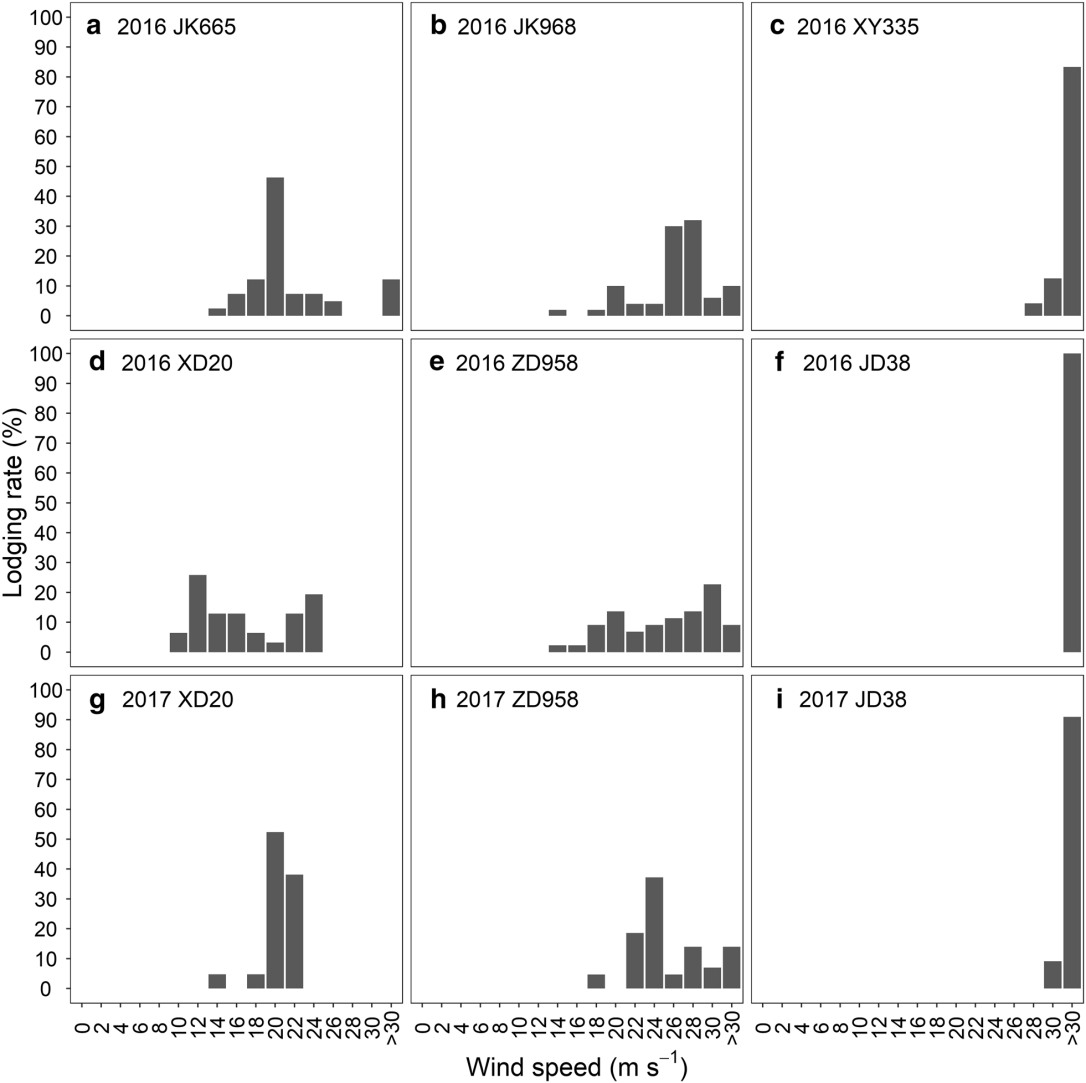
Lodging percentage at different wind speeds for six cultivars in 2016 (**a** for JK665, **b** for JK968, **c** for XY335, **d** for XD20, **e** for ZD958, and **f** for JD38) and three cultivars in 2017 (**g** for XD20, **h** for ZD958, and **i** for JD38). The lodging percentage at “> 30” means that none of the plants fell down even though a maximum wind speed of 30 m s^−1^ was generated in each experiment

Lodging cases in both 2016 and 2017 were all stalk lodging. The distribution of lodging percentage along wind speeds differed between cultivars and years (Fig. 3). The lodging percentage of XD20 and ZD958 showed bimodal distribution in 2016 but unimodal distribution in 2017. In 2016, JK665 and JK968 also showed unimodal distribution with a maximum lodging percentage of 46% at approximately 20 m s^−1^ and of 62% at wind speeds ranging from 24 to 28 m s^−1^, respectively. Two peak maxima of lodging percentage were observed at approximately 12 m s^−1^ and 24 m s^−1^ for XD20, and 20 m s^−1^ and 30 m s^−1^ for ZD958 in 2016. Very few of the plants for XY335 in 2016 and JD38 in both 2016 and 2017 broke until the maximum wind speed was applied, indicating a wind speed of higher than 30 m s^−1^ for stalk lodging to occur in XY335 and JD38.

**Fig. 4:**
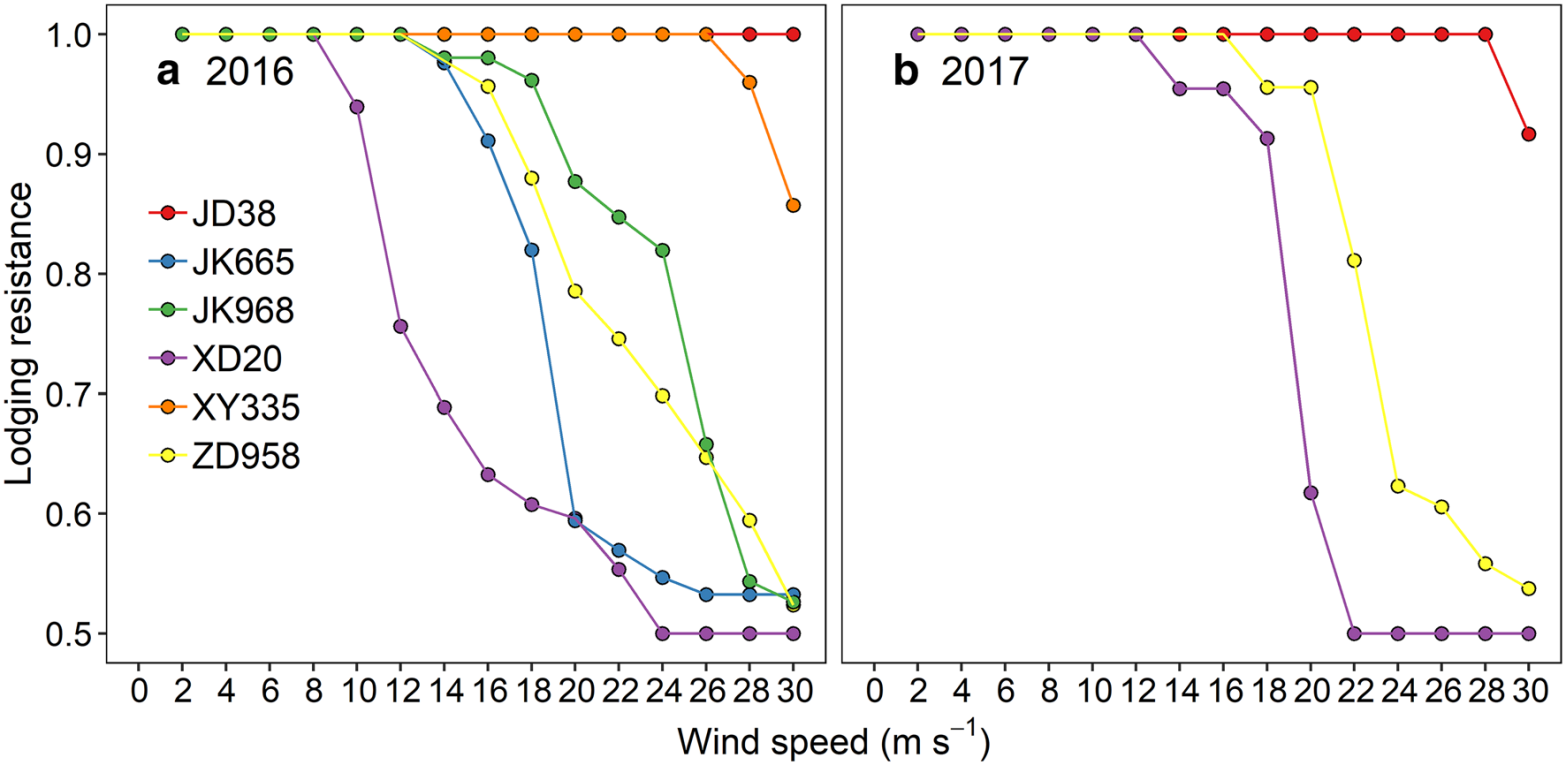
Quantified lodging resistance at different generated wind speeds for six cultivars in 2016 (**a**) and three cultivars 2017 (**b**). Red, blue, green, purple, orange and yellow symbols represent JD38, JK665, JK968, XD20, XY335 and ZD958 respectively

The FWSs of the cultivars were estimated using Eq. 2. Cultivar XD20 had the lowest FWS of 16 m s^−1^, while FWSs were higher than 30 m s^−1^ for both JD38 and XY335. The FWSs was higher than 30 m s^−1^ for JD38, 24 m s^−1^ for ZD958, and 20 m s^−1^ for XD20 in 2017, showing the same rank in descending order as 2016 with a good reliability (i.e. nRMSE of 10.76%) (Table 2). Lodging resistance at different wind speeds was calculated according to the distribution of lodging percentage using Eq. 3. Lodging resistance showed a decreasing trend with increasing wind speed across cultivars, except for JD38 in 2016 (Fig. 4). For JD38, lodging was not observed below the wind speed of 30 m s^−1^ (Fig. 3). Subsequently, the rank for the CLIs of all cultivars, calculated by Eq. 4, were JD38, XY335, JK968, ZD958, JK665, and XD20 in 2016 (Table 2) in descending order. In the 2017 experiments, JD38 had the highest CLI and XD20 had the lowest CLI, which is consistent with the results from 2016. All tests for CLI were found to have an excellent reliability (i.e. nRMSE of 5.38%) (Table 2).

**Table 2:** CLI and FWS for different cultivars in 2016 and 2017 and their reliability

Cultivars	FWSs			CLIs		
	2016	2017	nRMSE (%)	2016	2017	nRMSE (%)
ZD958	26	24	10.76	0.854	0.87	5.38
JD38	> 30	> 30		1	0.994	
XD20	16	20		0.718	0.796	
XY335	> 30	–		0.988	–	
JK665	20	–		0.801	–	
JK968	26	–		0.880	–

### Attenuation of wind speed by maize canopy

**Fig. 5:**
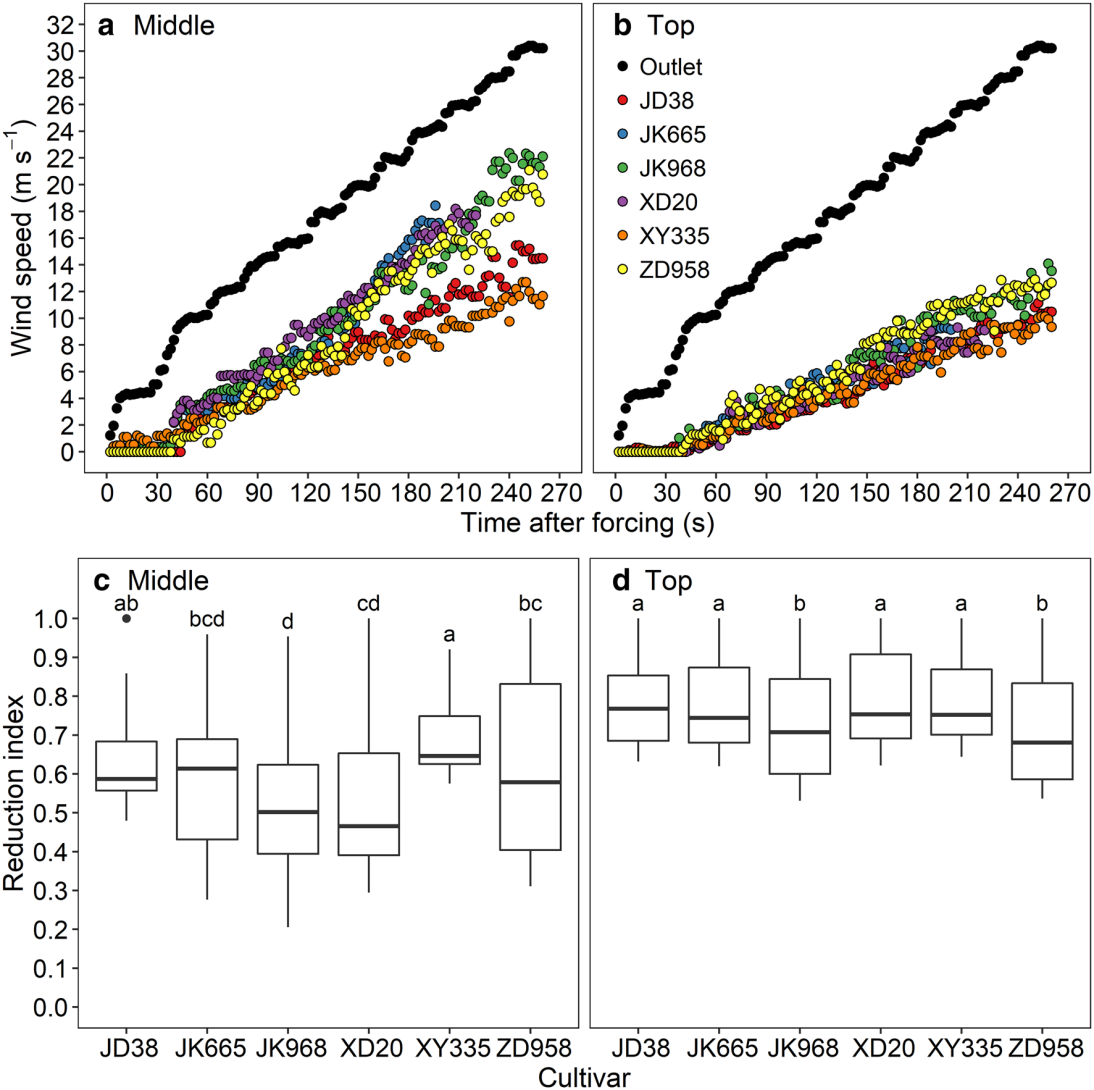
Measured wind speeds at the outlet of wind machine (black points in **a** and **b**), at the middle layer (**a**), and at the top layer of the holder (**b**) for different cultivars and the corresponding reduction index (**c** for the middle and **d** for the top). Red, blue, green, purple, orange and yellow represent JD38, JK665, JK968, XD20, XY335, and ZD958 respectively. Absence of shared letters denotes a statistically significant difference (P = 0.05) between cultivars

The wind speed at the outlet of the wind machine and at the end of the targeted rows increased with time after forcing following the pattern of wind regulation (Fig. 5). However, the slope of increasing trend at the end of the targeted rows differed between cultivars (Fig. 5). Initially, the dynamics of wind speed behind the rows were similar across cultivars until 150 s at which the generated wind speed was approximately 20 m s^−1^. Subsequently, the discrepancy between wind speed at the outlet and behind the rows was progressively greater for JD38 and XY335 due to the fact that most plants for these two cultivars remained standing even at the maximum generated wind speed of 30 m s^−1^. Significant differences in the reduction index of wind speed were found between cultivars at both middle and top layers. The *RI* of wind speed ranged from 0.53 to 0.69 at middle layers, and from 0.72 to 0.79 at top layer, indicating a lower reduction at the top layer due to the upward flow of wind. ZD958 and JK968 had significantly lower *RI* than other cultivars at the top layer. Among all of the cultivars XY335 and JD38 were found to have highest *RI* (Fig. 5).

### Relationship between CLI, stalk mechanical properties, and plant phenotypic traits

**Fig. 6:**
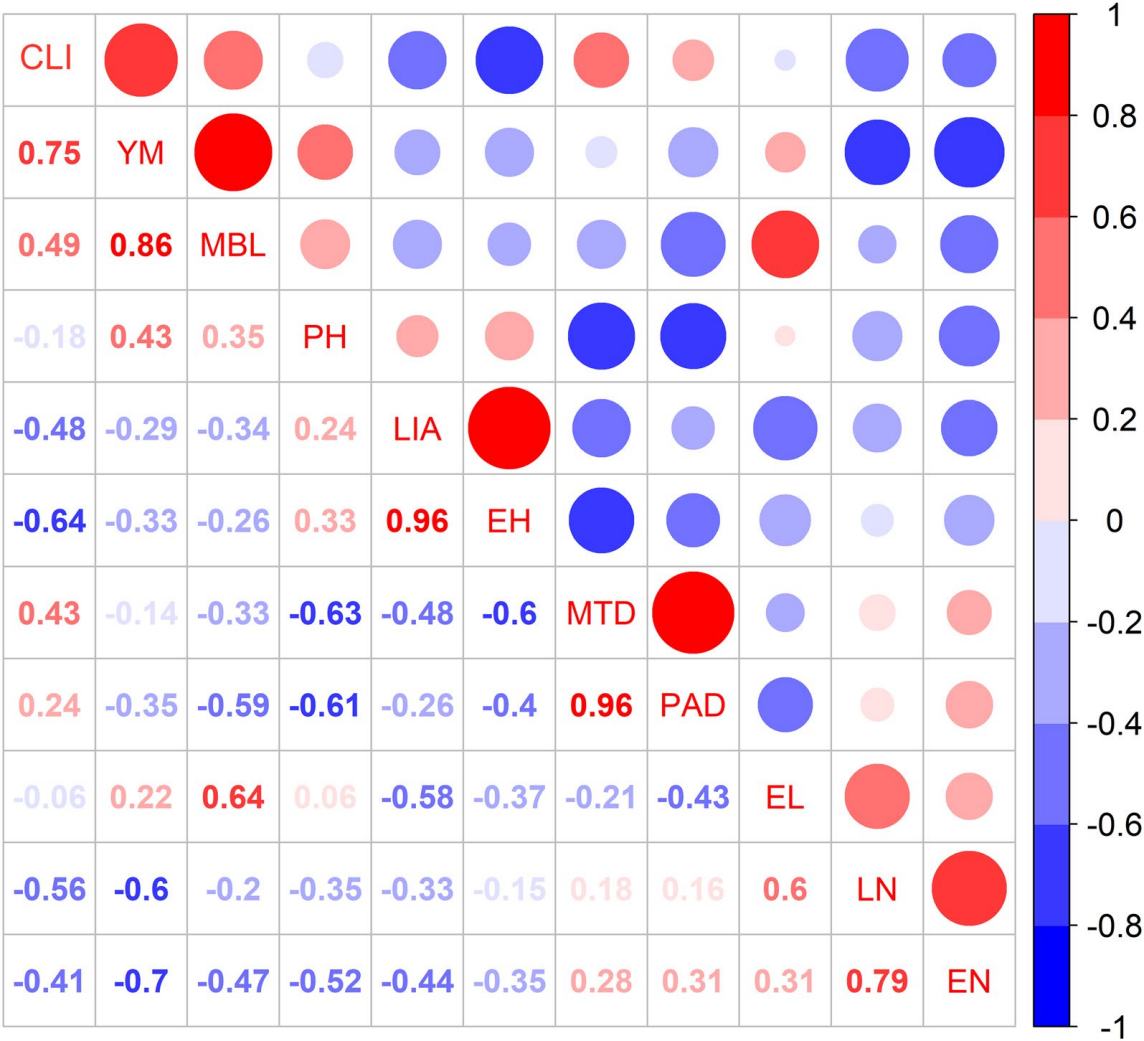
Correlation coefficients between CLI, mechanical properties and phenotypic traits. CLI, YM, MBL, PH, LIA, EH, MTD, PAD, EL, LN and EN represent cumulative lodging index, Young’s modulus, maximum bending load, plant height, leaf inclination angle, ear height, maximum transverse displacement, plant azimuthal deviation, ear length, leaf number, ear number

CLI was positively correlated with Young’s modulus, maximum bending load, and maximum transverse displacement with corresponding Pearson correlation coefficients of 0.75, 0.49, and 0.43, respectively, indicating the essential role of mechanical properties, especially Young’s modulus, in determining CLI (Fig. 6). Ear height, leaf number, and leaf inclination angle were the three factors that most negatively correlated with CLI with Pearson correlation coefficients of − 0.74, − 0.64, and − 0.56, respectively (Fig. 6). This means that increases of ear height, leaf number, and leaf inclination angle would increase the probability of stalk lodging in maize.

## Discussion

### FWS generated by lodging model, field investigation, and wind machine

This study showed that the mean FWS was greater than 22 m s^−1^ for different cultivars and years. This result is corroborated by previous estimation of 19 m s^−1^ via matching the maximum wind speed of extremely windy weather with the corresponding maize stalk lodging rate [29, 30]. Moreover, a lodging percentage of 20% was measured for XY335 and 5% for ZD958 under a condition with maximum wind speed of 21 m s^−1^ during the late growing season of maize [31], indicating that a FWS of higher than 21 m s^−1^ can be inferred for XY335 and ZD958. In addition, the FWS could be estimated between 11.6 and 22.8 m s^−1^ based on a theoretical model for the windthrow of plants [25], qualitatively confirming the FWS calculated by this study. However, the FWS of maize estimated by the lodging model and field investigation are lower than that by wind machine in the current study (Table 2). This discrepancy may be partly due to the fact that the failure is caused by successive gusts rather than by constant wind [32]. Furthermore, the progressive attenuation of the wind speed induced by the atmosphere resistance, which can be up to 50% (Fig. 2), was not reflected in this study.

### The difference between natural wind and simulated wind

Gusts of wind under natural conditions were found to represent a larger proportion of momentum in determining wind loading in comparison with that generated by the wind machine, indicating a difference in turbulence structure between these two cases [27]. Maize always fell massively under natural conditions [33] rather than as individuals, which is observed under wind machine tests. The discrepancy could be partly because winds would spread sideways to the nearby open areas under wind machine tests, whereas canopy turbulence is often generated under natural conditions [34]. Lodged maize plants were found to have a complete leaf shape in naturally windy conditions, whereas most of leaves were broken at the base of the sheath before stem lodging during wind tests. Dragging force of leaves, which largely depends on the leaf shape, leaf orientation, leaf fluttering, and turbulence [35], made a considerable contribution to stalk lodging [24]. The FWS might be overestimated in the present study due to partial absence of leaf dragging.

The turbulent nature of the wind could produce a rapidly fluctuating force on a plant in multiple directions. The plants in the middle of experimental rows were found to break first (unpublished data) due to the combination of strong wind from the tunnel and shaking airflow. Subsequently, neighboring plants lodged as a result of pressure from generated wind and fallen plants. The first two or three plants in the rows near the outlet always lodged last, remaining standing even under the maximum speed (unpublished data). Because the leaves of these plants were always damaged, the dragging force of leaves was largely reduced and they were not touched by nearby plants. Therefore, the deficiency in reproducing natural conditions could be one of the reasons for the discrepancy between the FWS from wind machine tests and that from multiple field investigations.

### Evaluation of crop lodging resistance

Mechanical properties, FWS, CLI, and RI were applied to evaluate maize lodging resistance. Mechanical properties, especially maximum bending load, were found to be negatively related to lodging percentage [36] and have been widely used as a comprehensive indicator in the evaluation of lodging resistance [14, 37]. None of the mechanical properties of the third internode for maize at R1 stage significantly differed between cultivars (Table 1). However, a previous study found that JD38 had greater maximum bending load of the third internode than JK968 at the V13 stage under the same conditions as this study [20]. Another study found that the maximum bending load differed significantly between ZD958 and XY335 in 2012, while not significantly in 2013 [31]. Therefore, the reliability of bending tests is poor, and the variation between cultivars is susceptible to be obscured [38].

FWS is an intuitive indicator to represent lodging resistance for plants, while plants do not always lodge at a specific wind speed (Fig. 3), impeding the accurate calculation of FWS. Accordingly, we applied *L*_1_ medians equation to determine FWS based on the distribution of lodging percentage (Table 2). However, distribution of lodging percentage varied substantially between years (Fig. 3) that the failure of maize plants for both the XD20 and ZD958 tended to occur at lower wind speeds in 2016 than that in 2017. This may be related to the variation in daily windspeed and rainfall during the week before wind machine tests. This also led to a low reliability for FWS (Table 2). As CLI normalized the lodging percentage at different wind speeds, it exhibited favorable reliability across cultivars and years (i.e., nRMSE of 5.38%). CLI also showed satisfactory resolution in evaluating lodging resistance between cultivars (Table 2). Although RI differed significantly and showed a similar trend with CLI between cultivars, it is a highly aggregated value that cannot separate the role of stalk lodging resistance in sheltering wind from that of plant morphology such as leaf geometry and internode size. Therefore, CLI is a more robust indicator to evaluate the lodging resistance in terms of both reliability and resolution.

### The influence of stalk mechanical properties and plant phenotyping traits

Maize lodging resistance can be improved by increasing mechanical properties and decreasing ear height, leaf number, and leaf inclination angle (Fig. 6). This is supported by previous results that the cell wall of mechanical tissue in the internode rind was found to be the most influential on maize stalk strength [39]. The stalk carbohydrate accumulation varies from R1 to mature, thus leading the changes of stalk mechanical properties [14]. Maize plants in full maturity, therefore, are expected to be more susceptible to stalk lodging than in R1 stage. In addition, ear height provides the arm of lodging force, which determines the moment of failure of a maize stem together with the lodging force [31]. Hence, ear height has been confirmed to be significantly correlated with maize lodging [14]. The negative effects of leaf number and leaf inclination angle in maize on stalk lodging resistance observed in our study were in agreement with the results reported by Sangoi et al. [40] and Zhang et al. [31]. A greater leaf number and leaf inclination angle would result in a higher dragging force, thus increasing the probability of lodging. This result provides a theoretical basis for screening lodging resistance phenotypes for maize breeding. No significant associations were found among CLI, mechanical properties, and plant phenotypic traits due to the limited samples of CLI in the experiments and inappropriate three-point bending test in which the span length is too short to produce the naturally occurring failure pattern in maize plants. The association analysis will be improved by accumulating additional experimental tests with keeping strictly identical managements in field trails between years, by increasing the number of plant phenotypes probably relate to stalk lodging, and by adopting improved three-point bending test for accurate phenotyping of maize stalk strength [41] in the future study.

### Potential implications for the future

A newly proposed method proved robust in quantifying stalk lodging resistance for different maize cultivars. However, FWS may not have been accurately determined for maize in this study because: (1) we were not able to mimic some characteristics of wind such as discrete gusts and lateral fluctuations that are present in nature due to constraining the flow direction by the wind machine; (2) we did not specify the aerodynamic parameters around the individuals when estimating FWS due to the lack of facilities to measure wind speed near the plants. As wind and lodging are intermittent for canopies [25], simulating different number of wind loading by wind machine may be more appropriate in the future. Computational fluid dynamics (CFD) simulating, a proven method in predicting airflow around both individuals and groups [42], could be used to predict wind reduction along the distance away from the wind machine to improve the accuracy of calculating FWS for maize. The crop lodging model developed by Baker [25] calculated the maximum wind at the moment of bending for an isolated plant, which was conceived as two simple masses connected by a weightless stem; this approach can also be applied for large populations of plants [43]. Hence, a lodging model calibrated for maize could help to identify FWS based on mechanical properties and plant parameters. Since incorrect measurement of aerodynamic and plant parameters could result in an over estimation of FWS by 51% [27], advanced tools such as laser scanning system [44], radar imaging [33], and high-speed video images [45, 46] could facilitate high-throughput measurement of and real-time access to parameters over the whole dragging process by wind. In addition, stem lodging resistance for maize was largely related to agronomic practices such as plant density [14] and plant growth regulator [14, 31]. Therefore, further evaluations of field lodging resistance should be conducted with more cultivars, varied plant densities, different growth periods, and a range of soil water conditions.

## Conclusions

This study characterized the pattern of lodging rate of maize in response to different wind speeds, and determine the FWS for different cultivars. A newly-built indicator, CLI, was demonstrated to be more robust in evaluating lodging resistance in terms of reliability and resolution than mechanical properties, FWS and RI. We proposed a straightforward approach to evaluating in situ lodging resistance of crops quantitatively. These results can also help breeders to identify lodging-resistant phenotypic traits accurately in future.

## Methods

### Field experiments

**Fig. 7:**
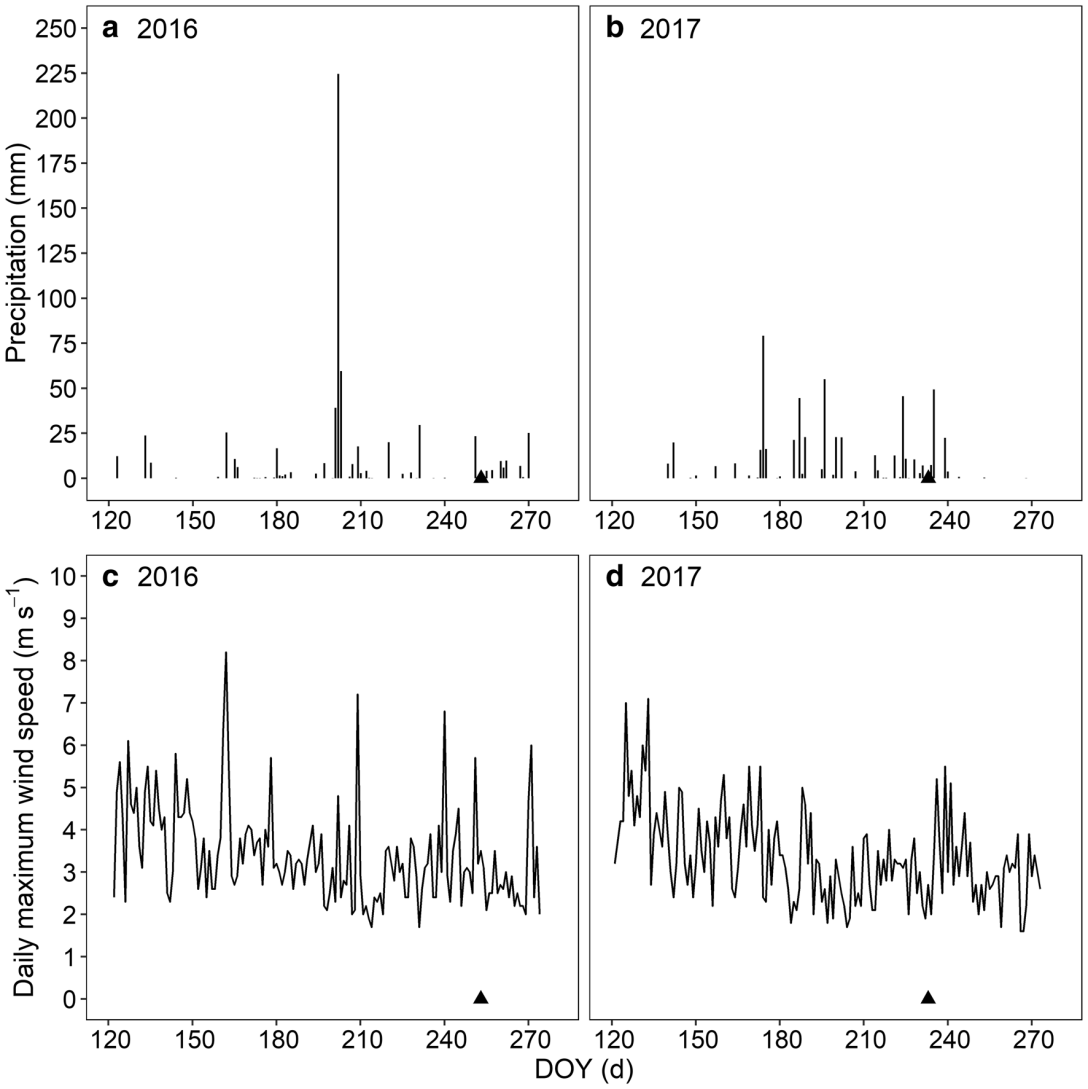
Precipitation (**a**, **b**) and daily maximum wind speed (**c**, **d**) during growing season in 2016 (**a**, **c**) and 2017 (**b**, **d**) in Haidian. The triangles denote the date when wind machine tests for maize were performed

Three experiments were performed in 2016 and 2017 at the *Beijing Academy of Agriculture and Forestry Sciences* (39°56′N, 116°16′E) in Beijing, China. The altitude of the study site is 70 m. The soil properties of the experimental field have been previously described by Zhang et al. [20]. Cultivation and management of the fields were the same in the three experiments. Maize was planted at a density of six plants m^−2^ with a row distance of 60 cm. Row orientation was south to north. All plots received compound fertilizer with 90 kg ha^−1^ N, 90 kg ha^−1^ P_2_O_5_, and 90 kg ha^−1^ K_2_O before sowing. Nitrogen fertilizer (135 kg ha^−1^) was applied on 12 July 2016 and on 20 July 2017. Crops were irrigated by spraying the field based on crop water requirements. Weeds were controlled regularly by hand. The precipitation and daily maximum wind speed during growing season in 2016 and 2017 are given in Fig. 7. The experimental field was exposed to neither heavy rain nor strong winds a week before wind machine tests in 2016 and 2017 (Fig. 7).

### Experiment 1: determining the effective range for the wind tunnel

In 2016, experiment 1 was performed before sowing to obtain the wind reduction caused by air resistance, thus determining the effective test area. The speed was altered approximately every 45 s referring to real-time wind speed measured at the middle of the outlet. Wind speeds were monitored at distances of 2, 4, 6, and 7 m away from the outlet of the wind machine using nine anemometers (HPT1000, Shiyutiancheng Co., Ltd.) fixed at three layers above the ground (0.2, 1.2, and 2.2 m). The wind speed data were separately averaged for the three layers above the ground to represent the wind speed at the corresponding level. The criteria for determining the effective area was that the wind speed at the specific distance away from the outlet under the generated maximum wind pressure should not be lower than the FWS of 19 m s^−1^, which was previously estimated [29, 30].

### Experiment 2: evaluating the lodging resistance of different cultivars

**Fig. 8:**
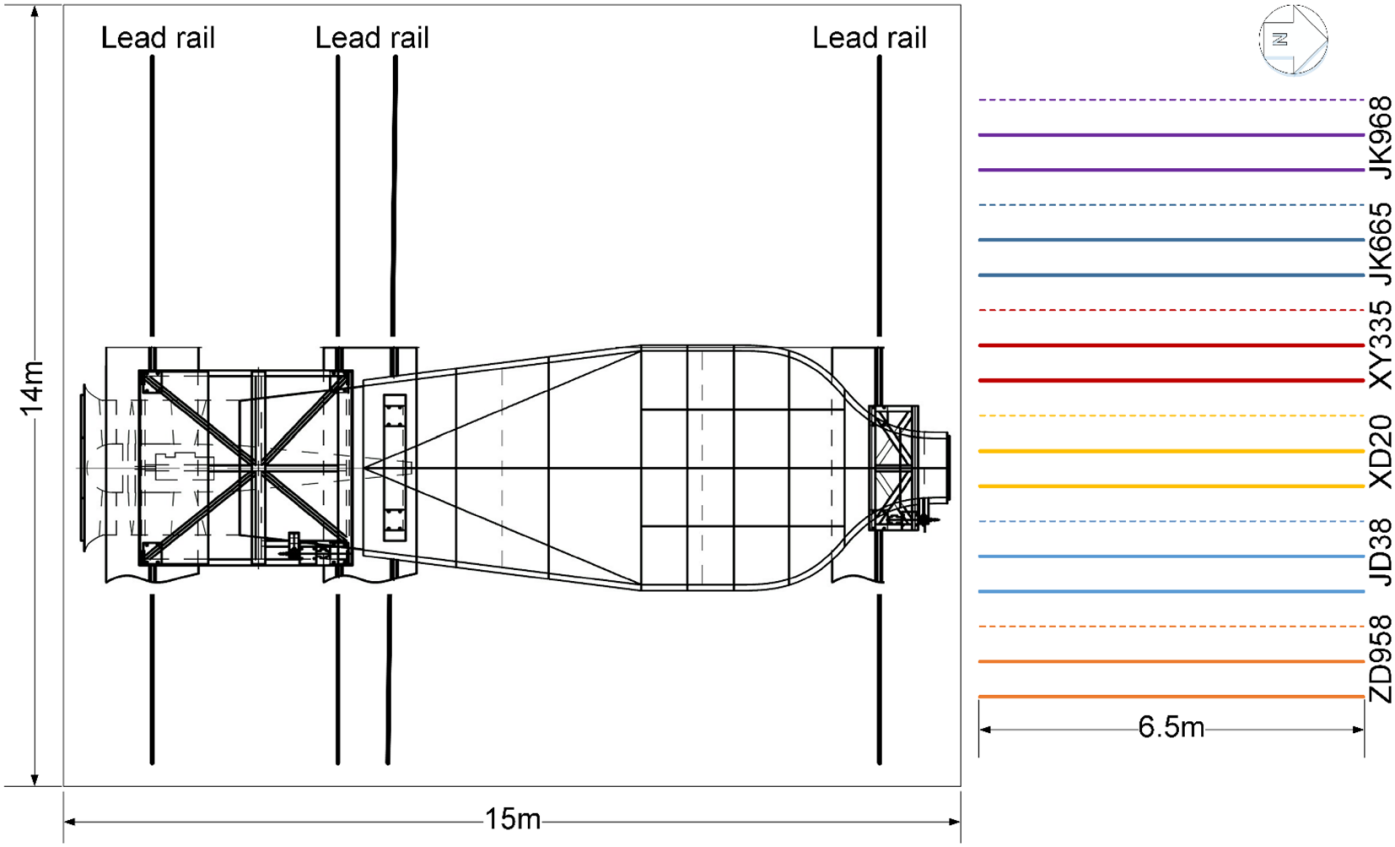
Schematic illustration of the experimental setup in 2016. Six cultivars, each of which had three rows, were planted with an identical row distance of 60 cm. Solid lines indicate two rows of maize for each cultivar that were tested and dashed lines indicate one row for backup and preventing targeted plants to be lodged during the test for the neighboring cultivar. The wind machine can be moved along west–east direction along four lead rails. Orange, blue, yellow, red, black and purple represent ZD958, JD38, XD20, XY335, JK665 and JK968

Experiment 2 was split into two sub-experiments: 2A and 2B. Experiment 2A was conducted in 2016 to evaluate the lodging resistance of different maize cultivars by applying mathematical models. Six maize cultivars with different stalk lodging resistances, Zhengdan958 (ZD958), Jingdan38 (JD38), Xundan20 (XD20), Xianyu335 (XY335), Jingke665 (JK665), and Jingke968 (JK968), were planted on 12 June 2016 in a rectangular plot (Fig. 8). Three consecutive rows for each maize cultivar were planted along the flow direction of the wind machine (Fig. 8). Experiment 2B was conducted in 2017 to evaluate the performance of the models. According to the results from experiment 2A, three cultivars, ZD958, JD38, and XD20, were selected and grown on 12 May 2017 in the same area. As maize is susceptible to late-season stalk lodging [38], wind machine tests were performed on 9 September 2016 in experiment 2A and on 21 August 2017 in experiment 2B when maize plants of all cultivars entered R1 stage.

**Fig. 9:**
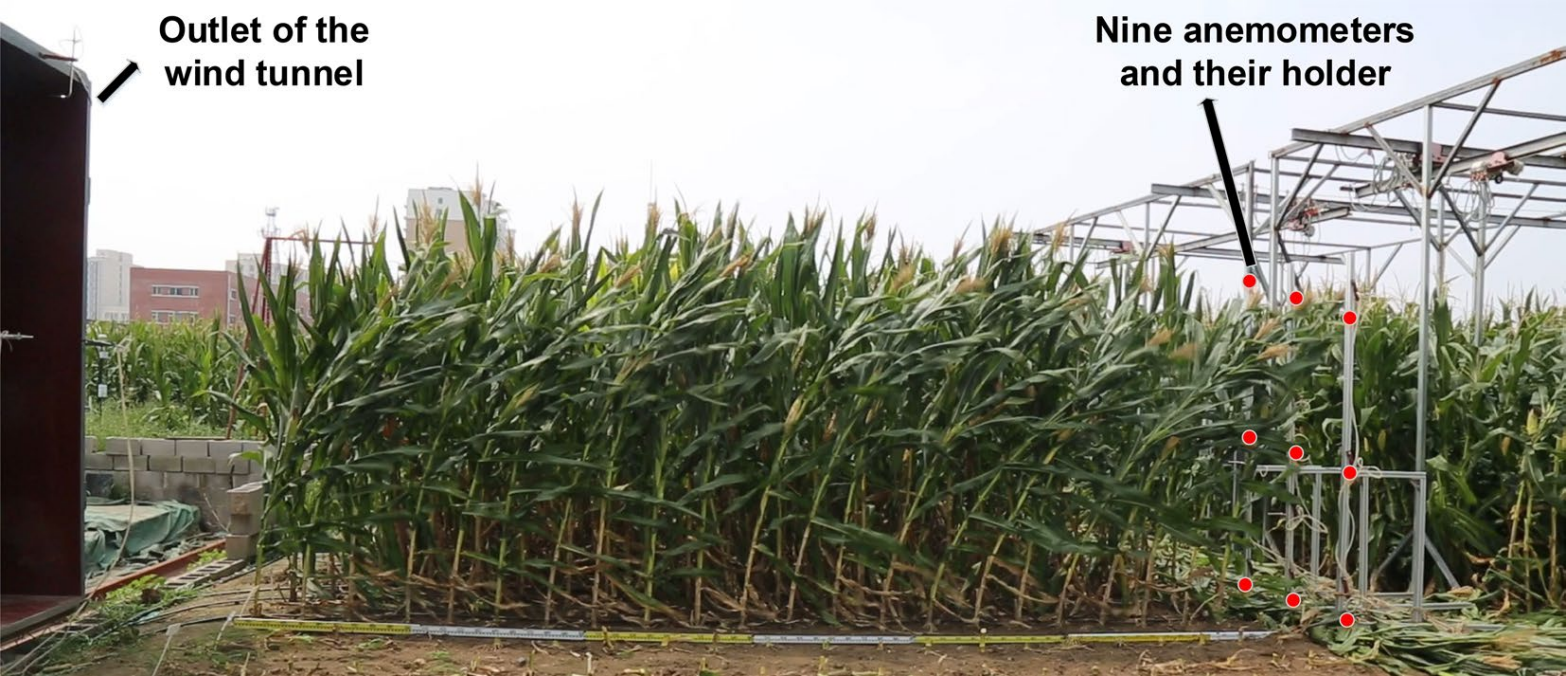
A photograph extracted from side-view video of an entire wind machine test for maize in field on 9 September 2016. Rows of maize for different cultivars were grown between the outlet of the wind machine and the holder for nine anemometers. Red dots indicate the positions of anemometers

For each wind machine experiment, two consecutive rows to the east of each plot were used (Fig. 8). The wind machine was moved to the place at which the middle of the outlet coincides with the middle of the selected consecutive row space. Different levels of wind conditions can be simulated by varying the fan speed based on the actual speed measurements at middle of the outlet (Fig. 9). The wind speed of the fan was increased in an interval of 5 m s^−1^ before reaching 10 m s^−1^, then the interval increased by 2 m s^−1^ until reaching the maximum speed of 30 m s^−1^. The wind speed was then gradually decreased to 0 m s^−1^. Each wind speed level was maintained for a period of 30 s before 10 m s^−1^ and 19 s from 10 to 30 m s^−1^. The wind speed has to be reduced gradually after all of the plants in the targeted rows succumbed to stem lodging. The anemometers were placed at the end of the targeted rows to capture the wind speed after sheltering by the maize canopy (Fig. 9). The number of lodged plants was counted as soon as the stalk failure occurred under the simulated wind conditions. Each test was recorded with side-view videos to validate the manual counting. When the test for a plot was finished, the wind machine and the anemometers were moved to the next plot and the above steps were followed. One of the tests is illustrated in Fig. 9.

### Experiment 3: measuring mechanical properties and phenotypic traits for different maize cultivars

Experiment 3 was laid adjacent (to the west of) to experiment 2A to the west in 2016 as a replicate for use in destructive measurements of stalk mechanical properties and plant phenotypic traits. Three plants were sampled randomly for each cultivar to measure mechanical properties of the internodes of rank 3, which were the most susceptible to late-season stalk lodging [47]. Another five plants were randomly sampled to measure phenotypic traits for each cultivar. The sampling work was performed on the same day as wind machine tests in 2016.

### Wind speed attenuation and reduction index

A reduction index was proposed to evaluate the ability of the objects (i.e. atmosphere and maize canopy) in reducing wind speed. Three anemometers were fixed at three layers, the bottom (0.2 m), middle (1.2 m), and top (2.2 m). The reduction index (*RI*_*s*_) at the generated wind speed of *s* (m s^−1^) was defined as:

1 $$RI_{s} = 1 - \frac{{\bar{u}_{1,s} }}{{\bar{u}_{0,s} }}$$

where $\bar{u}_{1,s}$ (m s^−1^) is the mean wind speed measured at the end of the targeted two rows and $\bar{u}_{0,s}$ (m s^−1^) is the speed at the outlet of the wind machine. *RI*_*s*_ indicates the average reduction in speed over the canopy due to the shelter provided by the maize canopy. A higher the index indicates that the maize cultivar may have greater lodging resistance.

### Measurement of stalk mechanical properties

Three-point bending tests were carries out in a laboratory setting to measure stalk mechanical properties using an Instron 3343 universal test machine (Instron 2519-104 Series, Norwood, Massachusetts, USA). Instron software (Bluehill 2.35) was used to control the equipment and record data. Stalk mechanical properties including Young’s modulus (MPa), maximum bending load (N), and maximum transverse displacement (mm), were measured according to the protocol outlined by Zhang et al. [20]. The segments used for the bending strength test were longer than 70 mm, which is the distance between the left and right supporting anvils of the test machine.

### Quantification of morphological traits of individual plants

Plant height, plant azimuthal deviation, leaf number, leaf inclination angle, ear number, ear height, and ear length were measured on five randomly selected plants. The plant azimuthal deviation describes the extent of leaves deviated from the azimuthal plane [48], which might affect the area exposed to the wind flow.

### Quantification of FWS and lodging resistance

FWS was regarded as a certain wind speed at which most of the plants at the targeted site fell down. To quantify the accurate FWS for the cultivars in specified environments, a mathematical expression was proposed according to our experiments. *FWS*_*c*_ was considered to be the wind speed at the global center along the distribution of lodging percentage for cultivar *c* and calculated using the *L*_1_ medians [49] as follows:

2 $$FWS_{c} = \mathop {\arg \hbox{min} }\limits_{{0 < s \le S_{m} }} \sum\limits_{v = 0}^{{S_{\text{max} } }} {p(v)\left| {v - s} \right|}$$

where *S*_max_ is the maximum wind speed that can be achieved in the wind machine tests, and *p*(*v*) is the lodging percentage.

Because all plants in the targeted site did not fall at a specific wind speed, the quantification of lodging resistance also depends on different wind speeds. FWS is not capable of describing the lodging resistance of cultivars at various wind speeds. Thus we proposed a new indicator, cumulative lodging index (CLI), to quantify the lodging resistance in relation to wind speeds. A higher percentage of falling plants at lower wind speeds indicates a low lodging resistance, and vice versa. The lodging resistance *LR*_*c*_(*s*) at a certain wind speed *s* was quantified as follows:

3 $$LR_{c} (s) = \frac{1}{{1 + \int_{0}^{s} {p(v){\text{d}}v} }}$$

where *p*(*v*) represents the percentage of plants that fell at wind speed *v*. The subscript *c* indicates the cultivar. According to the definition, *LR*_*c*_(*s*) is capable of characterizing the lodging resistance at any wind speed input *s*. Finally, a normalized area of lodging resistance function over a full range of wind speeds was defined as *CLI*_c_ for different cultivars. The *CLI*_c_ of cultivar *c* could be quantified as follows:

4 $$CLI_{c} = \int_{0}^{{S_{m} }} {LR_{c} (s){\text{d}}s} /S_{m}$$

### Statistical analysis

Multiple comparison of cultivars for mechanical properties, phenotypic traits, and reduction index were done by performing ANOVA (P = 0.05) analyses in the ‘stas’ package [50] and Tukey HSD tests (P = 0.05) in the ‘agricolae’ package [51] of the R programming language. To identify the major factors that contribute more to the lodging resistance, an association analysis was conducted using functions in the ‘corrplot’ package in R [52]. The reliability of the mathematical models was assessed using goodness of fit between the values of CLI and FWS in 2016 and in 2017, which was expressed as the normalized root mean square error (Nrmse, defined as RMSE divided by the average of the values in 2016) :

5 $$RMSE = \sqrt {\frac{1}{n}\sum\limits_{i = 1}^{n} {\left( {X_{2017,i} - X_{2016,i} } \right)^{2} } }$$

6 $$nRMSE = \frac{RMSE}{{\frac{1}{n}\sum\nolimits_{i = 1}^{n} {X_{2016,i} } }}$$

where *i* is the number of cultivars, *n* is the total number of cultivars, *X*_2016,*i*_ is the value in 2016 and the *X*_2017,*i*_ is the value in 2017. The model reliability is considered excellent with nRMSE < 10%, good if 10–20%, acceptable if 20–30% and poor if > 30% [53] shows best reliability when RMSE and nRMSE are close to 0.

## Additional file

**Additional file 1.** A video of test on maize plants of XD20 in 2016 using the wind machine.

## Data Availability

The datasets used and/or analyzed during the current study are available from the corresponding author on reasonable request.

## Abbreviations

EH: ear height (mm)
EL: ear length (mm)
EN: ear number
FWS: failure wind speed (m s^−1^)
CLI: cumulative lodging index (unitless)
RP: reliability of test
LIA: leaf inclination angle (°)
LN: leaf number
*LR*_*c*_(*s*): the lodging resistance at a certain wind speed *s* for the cultivar *c* (unitless)
MBL: maximum bending load (N)
PAD: plant azimuthal deviation (°)
PH: plant height (mm)
*RI*_*s*_: reduction index at the generated wind speed of *s* (unitless)
YM: Young’s modulus (MPa)
